# Supplementary material for: Resolving the full spectrum of human genome variation using Linked-Reads
Source: Genome Res. 2019 Apr;29(4):635–45. doi: 10.1101/gr.234443.118 (PMC6442396; doi:10.1101/gr.234443.118)
Supplement: Supplemental Material [file supp_gr.234443.118_Supplemental_longranger-2.2.zip › longranger-2.2.tar.bz2!/lr2.2-public/lib/stan/cmdstan/stan/doc/api/html/agrad_2rev_2functions_8hpp_source.html]

Stan: src/stan/agrad/rev/functions.hpp Source File


|  |  |
| --- | --- |
|  | Stan  2.5.0  probability, sampling & optimization |


- Main Page
- Related Pages
- Namespaces
- Classes
- Files

- File List
- File Members

All Classes Namespaces Files Functions Variables Typedefs Enumerations Enumerator Friends Macros Pages

- src
- stan
- agrad
- rev

functions.hpp

Go to the documentation of this file.

1 #ifndef STAN\_\_AGRAD\_\_REV\_\_FUNCTIONS\_HPP

2 #define STAN\_\_AGRAD\_\_REV\_\_FUNCTIONS\_HPP

3

4 #include <stan/agrad/rev/functions/Phi.hpp>

5 #include <stan/agrad/rev/functions/Phi\_approx.hpp>

6 #include <stan/agrad/rev/functions/abs.hpp>

7 #include <stan/agrad/rev/functions/acos.hpp>

8 #include <stan/agrad/rev/functions/acosh.hpp>

9 #include <stan/agrad/rev/functions/as\_bool.hpp>

10 #include <stan/agrad/rev/functions/asin.hpp>

11 #include <stan/agrad/rev/functions/asinh.hpp>

12 #include <stan/agrad/rev/functions/atan.hpp>

13 #include <stan/agrad/rev/functions/atan2.hpp>

14 #include <stan/agrad/rev/functions/atanh.hpp>

15 #include <stan/agrad/rev/functions/bessel\_first\_kind.hpp>

16 #include <stan/agrad/rev/functions/bessel\_second\_kind.hpp>

17 #include <stan/agrad/rev/functions/binary\_log\_loss.hpp>

18 #include <stan/agrad/rev/functions/cbrt.hpp>

19 #include <stan/agrad/rev/functions/ceil.hpp>

20 #include <stan/agrad/rev/functions/cos.hpp>

21 #include <stan/agrad/rev/functions/cosh.hpp>

22 #include <stan/agrad/rev/functions/digamma.hpp>

23 #include <stan/agrad/rev/functions/erf.hpp>

24 #include <stan/agrad/rev/functions/erfc.hpp>

25 #include <stan/agrad/rev/functions/exp.hpp>

26 #include <stan/agrad/rev/functions/exp2.hpp>

27 #include <stan/agrad/rev/functions/expm1.hpp>

28 #include <stan/agrad/rev/functions/fabs.hpp>

29 #include <stan/agrad/rev/functions/falling\_factorial.hpp>

30 #include <stan/agrad/rev/functions/fdim.hpp>

31 #include <stan/agrad/rev/functions/floor.hpp>

32 #include <stan/agrad/rev/functions/fma.hpp>

33 #include <stan/agrad/rev/functions/fmax.hpp>

34 #include <stan/agrad/rev/functions/fmin.hpp>

35 #include <stan/agrad/rev/functions/fmod.hpp>

36 #include <stan/agrad/rev/functions/gamma\_p.hpp>

37 #include <stan/agrad/rev/functions/gamma\_q.hpp>

38 #include <stan/agrad/rev/functions/hypot.hpp>

39 #include <stan/agrad/rev/functions/ibeta.hpp>

40 #include <stan/agrad/rev/functions/if\_else.hpp>

41 #include <stan/agrad/rev/functions/inv.hpp>

42 #include <stan/agrad/rev/functions/inv\_cloglog.hpp>

43 #include <stan/agrad/rev/functions/inv\_logit.hpp>

44 #include <stan/agrad/rev/functions/inv\_sqrt.hpp>

45 #include <stan/agrad/rev/functions/inv\_square.hpp>

46 #include <stan/agrad/rev/functions/is\_inf.hpp>

47 #include <stan/agrad/rev/functions/is\_nan.hpp>

48 #include <stan/agrad/rev/functions/is\_uninitialized.hpp>

49 #include <stan/agrad/rev/functions/lgamma.hpp>

50 #include <stan/agrad/rev/functions/lmgamma.hpp>

51 #include <stan/agrad/rev/functions/log.hpp>

52 #include <stan/agrad/rev/functions/log10.hpp>

53 #include <stan/agrad/rev/functions/log1m.hpp>

54 #include <stan/agrad/rev/functions/log1m\_exp.hpp>

55 #include <stan/agrad/rev/functions/log1p.hpp>

56 #include <stan/agrad/rev/functions/log1p\_exp.hpp>

57 #include <stan/agrad/rev/functions/log2.hpp>

58 #include <stan/agrad/rev/functions/log\_diff\_exp.hpp>

59 #include <stan/agrad/rev/functions/log\_falling\_factorial.hpp>

60 #include <stan/agrad/rev/functions/log\_rising\_factorial.hpp>

61 #include <stan/agrad/rev/functions/log\_sum\_exp.hpp>

62 #include <stan/agrad/rev/functions/modified\_bessel\_first\_kind.hpp>

63 #include <stan/agrad/rev/functions/modified\_bessel\_second\_kind.hpp>

64 #include <stan/agrad/rev/functions/multiply\_log.hpp>

65 #include <stan/agrad/rev/functions/owens\_t.hpp>

66 #include <stan/agrad/rev/functions/pow.hpp>

67 #include <stan/agrad/rev/functions/primitive\_value.hpp>

68 #include <stan/agrad/rev/functions/rising\_factorial.hpp>

69 #include <stan/agrad/rev/functions/round.hpp>

70 #include <stan/agrad/rev/functions/sin.hpp>

71 #include <stan/agrad/rev/functions/sinh.hpp>

72 #include <stan/agrad/rev/functions/sqrt.hpp>

73 #include <stan/agrad/rev/functions/square.hpp>

74 #include <stan/agrad/rev/functions/step.hpp>

75 #include <stan/agrad/rev/functions/tan.hpp>

76 #include <stan/agrad/rev/functions/tanh.hpp>

77 #include <stan/agrad/rev/functions/tgamma.hpp>

78 #include <stan/agrad/rev/functions/trunc.hpp>

79 #include <stan/agrad/rev/functions/value\_of.hpp>

80

81 #endif

fabs.hpp

gamma\_p.hpp

hypot.hpp

cbrt.hpp

owens\_t.hpp

log.hpp

fma.hpp

modified\_bessel\_second\_kind.hpp

inv\_logit.hpp

log\_rising\_factorial.hpp

gamma\_q.hpp

inv.hpp

log\_falling\_factorial.hpp

modified\_bessel\_first\_kind.hpp

ceil.hpp

sin.hpp

tanh.hpp

primitive\_value.hpp

binary\_log\_loss.hpp

Phi\_approx.hpp

ibeta.hpp

erfc.hpp

exp2.hpp

log10.hpp

exp.hpp

inv\_square.hpp

floor.hpp

tan.hpp

asin.hpp

inv\_sqrt.hpp

bessel\_second\_kind.hpp

is\_nan.hpp

if\_else.hpp

atanh.hpp

round.hpp

is\_uninitialized.hpp

acos.hpp

falling\_factorial.hpp

log\_sum\_exp.hpp

Phi.hpp

lmgamma.hpp

atan.hpp

fmax.hpp

cos.hpp

fmod.hpp

log1m.hpp

log1p\_exp.hpp

lgamma.hpp

sqrt.hpp

tgamma.hpp

bessel\_first\_kind.hpp

erf.hpp

is\_inf.hpp

atan2.hpp

square.hpp

cosh.hpp

multiply\_log.hpp

inv\_cloglog.hpp

as\_bool.hpp

expm1.hpp

log1m\_exp.hpp

acosh.hpp

abs.hpp

trunc.hpp

log2.hpp

fdim.hpp

sinh.hpp

log\_diff\_exp.hpp

asinh.hpp

digamma.hpp

step.hpp

rising\_factorial.hpp

fmin.hpp

value\_of.hpp

pow.hpp

log1p.hpp


---

[ Stan Home Page ]

*© 2011–2014,
Stan Development Team.*
